# Developmentally regulated generation of a systemic signal for long‐lasting defence priming in tomato

**DOI:** 10.1111/nph.20288

**Published:** 2024-11-19

**Authors:** Katie Stevens, Michael R. Roberts, Katie Jeynes‐Cupper, Lamya Majeed, Victoria Pastor, Marco Catoni, Estrella Luna

**Affiliations:** ^1^ School of Biosciences University of Birmingham Birmingham B15 2TT UK; ^2^ Lancaster Environment Centre Lancaster University Lancaster LA1 4YQ UK; ^3^ Plant Immunity and Biochemistry Laboratory, Biology, Biochemistry and Natural Sciences Department University Jaume I 12071 Castellon Spain; ^4^ Present address: Department of Plant Breeding Swedish University of Agricultural Sciences 234 56 Alnarp Sweden

**Keywords:** *Botrytis cinerea*, DNA methylation, grafting, postharvest, sRNA, β‐aminobutyric acid

## Abstract

- Tomato is a major global crop. However, its production is limited by *Botrytis cinerea.* Due to the toxicity of postharvest pesticide application, alternative control methods such as priming are being investigated.
- Plants were treated with β‐aminobutyric acid (BABA) at two developmental stages and resistance against *B. cinerea* was tested in fruit tissue and in progenies. DNA methylation and RNA sequencing were conducted to characterise the (epi)genetic changes associated with long‐lasting resistance. Grafting experiments were done to assess the systemic nature of this signal, which was further characterised by small RNA (sRNA) sequencing of scions.
- Only BABA‐treated seedlings displayed induced resistance (IR). DNA methylation analysis revealed seedling‐specific changes, which occurred in the context of lower basal methylation. BABA‐IR was found to be transmissible from primed rootstock to grafted unprimed scions. In these scions, we identified a subset of mobile 24 nt sRNAs associated with genes showing primed expression during infection in fruit.
- Our results demonstrate the functional association of a systemic signal with long‐lasting IR and priming. Through integrated omics approaches, we have identified markers of long‐lasting priming in tomato fruit which could also serve as targets for durable resistance in other crops.

## Introduction

The current food supply chain experiences major losses at the postharvest level due to both injury and infection by pathogenic fungi (Lipinski *et al*., 2013; Zhang *et al*., 2021). Tomato is a major global commodity, with 182.3 million tons of fruit produced in 2019 (FAO, 2019). However, its yield is heavily restricted due to pathogens, and 50% of yield loss occurs at the postharvest stage (FAO, 2019). Postharvest pesticide use is not permitted for tomato fruit in commercial settings (Pétriacq *et al*., 2018), and the main control measures at this stage are limited to cold temperature storage and strict hygiene measures (Abbey *et al*., 2019). However, postharvest pathogens such as *Botrytis cinerea*, the causal agent of grey mould, cannot be successfully controlled with these strategies. Therefore, new approaches are required. A better understanding of tomato defence mechanisms would allow researchers to design strategies to control pre‐ and postharvest fungal infections and reduce yield waste.

The ‘adaptive’ component of the plant immune system can be referred to as priming (Mauch‐Mani *et al*., 2017). Unlike direct activation of defence mechanisms, which induces significant metabolic alterations, priming minimises energetic costs via targeted allocation of energy resources upon attack, thus resulting in a faster and stronger activation of defence mechanisms when required (van Hulten *et al*., 2006). Priming is considered to be broad spectrum and has been described in many different plant species, from *Arabidopsis thaliana* to *Malus pumila* (apple trees) (Zimmerli *et al*., 2000; Cohen, 2002; Reuveni *et al*., 2003; Cohen *et al*., 2010, 2016). Importantly, priming has been shown to be long‐lasting (Worrall *et al*., 2012; Wilkinson *et al*., 2018; Mageroy *et al*., 2020; Catoni *et al*., 2022) and to be transmitted to following generations (Luna *et al*., 2011; Slaughter *et al*., 2011; Rasmann *et al*., 2012). A very well‐characterised priming chemical is the nonprotein amino acid β‐aminobutyric acid (BABA), first identified in the 1960s (Papavizas & Davey, 1963). BABA has subsequently been documented to be effective against both abiotic and biotic stresses in a range of species (Cohen *et al*., 2016). BABA‐induced resistance (BABA‐IR) is associated with a range of changes to the plant such as enhanced physical protection through callose deposition, PATHOGENESIS‐RELATED1 (PR1) protein accumulation and increases in defence hormones such as salicylic acid (SA) and jasmonic acid (JA) (Zimmerli *et al*., 2000; Ton & Mauch‐Mani, 2004; Hamiduzzaman *et al*., 2005; Ton *et al*., 2005; Schwarzenbacher *et al*., 2020). In Arabidopsis, BABA binds to an aspartyl‐tRNA synthetase (Luna *et al*., 2014) and changes the canonical function of the enzyme into priming. In tomato and Arabidopsis, BABA can be absorbed through the roots and is then translocated to aerial tissue (Cohen & Gisi, 1994; Wilkinson *et al*., 2018). Although the receptor has not been identified in tomato, BABA is thought to work in a similar way in tomato to Arabidopsis (Luna *et al*., 2014), leading to durable enhanced resistance against *B. cinerea* (Luna *et al*., 2016). BABA treatment has been shown to lead to long‐lasting protection of fruit tissue when applied at the seedling stage, thus conferring postharvest protection (Wilkinson *et al*., 2018; Luna *et al*., 2020). Therefore, long‐lasting priming offers an alternative approach to fungicides towards protecting plants from postharvest pathogenic infections.

Long‐lasting priming has been linked to epigenetic changes such as DNA methylation and the production of small RNAs (sRNAs), as they can contribute to changes in gene expression (Slaughter *et al*., 2011; Dowen *et al*., 2012; Rasmann *et al*., 2012; Catoni *et al*., 2022; Hannan Parker *et al*., 2022). For instance, analysis of Arabidopsis epigenetic recombinant inbred lines (epiRIL) demonstrated that hypomethylated loci enhanced priming of SA‐dependent and SA‐independent defences against virulent *Hyaloperonospora arabidopsidis* (Furci *et al*., 2019). Moreover, sRNAs produced by the plant‐specific RNA‐directed DNA methylation (RdDM) pathway have been associated with long‐lasting and transgenerational IR in Arabidopsis (Rasmann *et al*., 2012). Recent work has illustrated that JA‐IR is regulated by DNA‐demethylation pathways, requiring an intact sRNA binding protein AGO1 to prime defence‐associated genes (Wilkinson *et al*., 2023). BABA has also been shown to be associated with important changes in DNA methylation. In tomato, global changes to DNA methylation in the CHH cytosine context (H indicates any nucleotide other than G) have been associated with long‐lasting BABA‐IR in the Money‐Maker cultivar. While many differentially methylated regions (DMRs) were found in promoters of differentially expressed genes (DEGs) during *B. cinerea* infection, the majority of primed genes were not differentially methylated (Catoni *et al*., 2022). Therefore, the mechanisms behind the long‐lasting epigenetic nature of priming are still unclear. In addition, the long‐lasting nature of BABA‐IR has yet to be explored and utilised for its potential role in postharvest resistance. Interestingly, tomato plants have been shown to have different methylation profiles depending on both fruit developmental stage and tissue type: CG and CHG methylation levels are lower in fruit tissue than in 4‐wk‐old leaf tissue, with the reverse pattern seen in CHH context (Zhong *et al*., 2013). However, how changes in developmental stage‐dependent DNA methylation mediate the imprinting and the maintenance of long‐lasting postharvest priming is unexplored.

Here, we found that the plant's developmental stage has a major influence on the ability to establish long‐lasting priming against *B. cinerea*. We assessed the impact of BABA treatments on a transcriptomic and epigenomic level at different developmental stages and used methylome analysis to test the hypothesis that young plants display greater epigenetic plasticity. Additionally, we found that long‐lasting BABA‐IR is transmissible to naive scion tissue when grafted on primed rootstock, and we investigated the association of sRNAs with resistance. Through the integration of omics analyses, we have identified markers associated with long‐lasting BABA‐IR in tomato for the control of *B. cinerea* in fruit postharvest.

## Materials and Methods

### Plant materials and growth conditions

Seeds of tomato cv Micro Tom (*Solanum lycopersicum* L.) were placed in Petri dishes containing wetted tissue paper and maintained at 28°C in the dark for 3–6 d to stimulate homogeneous germination. Germinated seeds were planted in individual pot propagators containing Scott's M3 compost and cultivated under standard tomato growth conditions (16 h : 8 h, day : night cycle; 25°C : 20°C).

### BABA treatments and sample collection

At 2 wk, one‐third of the plants were soil drenched with 5 mM BABA (catalogue no.: A4420‐7; Sigma Aldrich) to provide a final concentration of 0.5 mM in the soil. All other plants were treated with the same volume of water. One week after BABA treatment, seedlings were removed from soil, roots were gently washed, and seedlings were replanted in new compost. At 12 wk, one‐third of the plants were soil‐treated to a final concentration of 0.5 mM BABA. The remaining one‐third of the plants were left as water controls. One week after each respective BABA treatment, two leaves from three individual plants were collected and stored in liquid nitrogen. Plants were left to grow and produce red fruit. The first three fruits on all plants were marked.

### Relative growth rate analysis

Plant height was measured before BABA treatment at 2 wk (t1) and 1 wk after BABA treatment (t2), as previously described (Luna *et al*., 2016). Briefly, height was measured with a ruler from the soil to the junction of the newest leaves. Relative growth rate (RGR) was calculated using the formula = LN(height − t2) − LN(height − t1)/t2 − t1 (Pérez‐Harguindeguy *et al*., 2013).

### *Botrytis cinerea* infections

Cultivation of *Botrytis cinerea* was performed as described in Luna *et al*. (2016); briefly, the pathogen was grown on PDA plates for 4 wk in the dark. Infections of leaves with *Botrytis cinerea* (R16) were performed entirely as described previously (Luna *et al*., 2016). Briefly, detached leaves were inoculated with 5 μl drops containing an inoculum of 1 × 10^6^ spores ml^−1^. Infected leaves were kept at 100% humidity and in the dark for the duration of the experiment. Disease was scored by measuring lesion diameters. Infection of fruit was performed entirely as described in Wilkinson *et al*. (2018). Briefly, ripened tomatoes were placed in quail egg boxes with the tip pointing upwards and pierced before inoculation with a 5 μl drop of 5 × 10^4^ spores ml^−1^. After inoculation, tomatoes were kept at 100% humidity in the dark for the duration of the experiment. Disease was scored by classifying fruit in different categories of infection and distributions were represented as disease severity index as described previously (Wilkinson *et al*., 2018). Statistical analysis was done in R (v.1.4.1717) and as previously described (Luna *et al*., 2016; Wilkinson *et al*., 2018).

### Nucleic acid extractions

Leaf material from three independent plants was collected 1 wk after BABA treatment. At 15 wk, the first three fruits were collected from each plant and stored in liquid nitrogen for DNA extraction for methylation analysis. DNA was extracted from 100 mg of ground frozen leaf and fruit tissue using Qiagen DNeasy Plant Mini Kit (Qiagen) following the manufacturer's instructions and resuspended in 30 μl H_2_O. For transcriptomic analysis, leaf and fruit tissue from three plants was collected at 1 and 2 d postinoculation (dpi), respectively. RNA for all experiments was extracted from 100 mg of ground frozen tissue using TRIzol (catalogue no.: 15596026; Thermo Fisher, Waltham, MA, USA) following the manufacturer's specifications.

### Library preparation and sequencing mRNA data

mRNA sequencing analysis was conducted on RNA extracted from fruit tissue 48 h postinfection (hpi) with *B. cinerea*. Library preparation and sequencing were conducted by Novogene using NovaSeq 6000 PE150. Twenty million paired reads were generated per sample. A total of 767 502 726 clean reads were generated across 17 samples, with an average of 45 147 219 clean reads per sample. An average of 92.6% of nucleotides per sample had a Phred quality score of > 30. The quality of samples was assessed using Fast qc (v.0.11.5‐Java‐1.8.0_74). Adapters were removed from samples using Trimmomatic (v.0.39) (Bolger *et al*., 2014). Reads were aligned to the tomato genome (Itag4.0) using Hisat2 (v.2.2.1) (Kim *et al*., 2019). Samtools (v.1.12) (Li *et al*., 2009) was used to sort and index Sam files into Bam format. Read counts were generated using Htseq (v.0.13.5) (Anders *et al*., 2015). All sequencing information is summarised in Supporting Information Table S1.

### Statistical analysis of sequence count files

Count files were analysed in R using deseq2 (v.1.34.0) (Love *et al*., 2014). Genes with total read counts of under 10 were removed. A variance stabilising transformation (VST) was applied for normalisation, principal component analysis (PCA) plots were generated using deseq2 and ggplot2, and hierarchical clustering was conducted for samples using Euclidean distances. Differentially expressed genes involved in infection were identified for infected plants of all three conditions (BABA2, Water and BABA12). DEGs were identified between BABA2 and water, and between BABA12 and water plants to identify the effect of early and late BABA treatments on the transcriptome without infection.

### Library preparation and whole‐genome bisulfite sequencing processing

All library preparation and sequencing were conducted by Novogene using NovaSeq 6000 PE150. After quality control, positive control DNAs were added into the DNAs which were then fragmented into 200–400 bp using Covaris S220 and bisulfite treated (Accel‐NGS Methyl‐Seq DNA Library Kit for illumina, Swift). Ligation of methylation sequencing adapters, size selection and PCR amplification was then performed before illumina sequencing for 20 million paired reads per sample with 30× sequencing depth. An average of 90.2% of nucleotides per sample had a Phred quality score of > 30. A total of 1.1 × 10^9^ reads were generated across all 24 samples, with an average of 45.8 million clean reads per sample. The tomato reference genome (v.4.0) was merged with the chloroplast sequence (NC_007898.3) to calculate the bisulfite conversion rate. The reference genome was bisulfite converted using Bismark (v.0.16.3_bowtie2) (Krueger & Andrews, 2011). Quality of samples was assessed using Fast qc (v.0.11.5‐Java‐1.8.0_74). Adapters were removed from samples using Trimmomatic (v.0.39) (Bolger *et al*., 2014). Reads were aligned to the SL4.0 tomato genome using Bismark (Krueger & Andrews, 2011). The average mapping efficiency was 80% with a minimum and maximum value of 77% and 82.2%, respectively. Samples were de‐duplicated using Samtools (/1.8‐iomkl‐2018a). The bisulfite conversion rate was calculated for each sample using the chloroplast sequence; conversion rates varied from 98.41% to 99.66%. An estimated conversion rate was applied following Catoni & Zabet (2021) in order to account for nonconverted DNA. All sequencing information is summarised in Table S1.

### Analysis of DNA methylation

Differentially methylated regions were identified starting from the Bismark CX files using a previously established pipeline (Catoni & Zabet, 2021). Briefly, we used the package *DMRcaller* (v.1.26.0) (Catoni *et al*., 2018) to identify DMRs in each cytosine context (CG, CHG and CHH) in all paired combinations of our samples, including three different timepoints: T1 (3 wk) Water and BABA2 leaf tissue 1 wk after BABA soil drench; T2 (13 wk) Water, BABA2 and BABA12 leaf tissue 1 wk after BABA soil drench; and T3 (15 wk) Ripe red fruit in water, BABA2 and BABA12. The following parameters were used: function *computeDMRsReplicates*, the ‘bins’ method was used with a bin size of 200 bp, methylationDiff of 0.2, minCytosinesCount of 2, minReadsPerCytosine of 4 and a minimum *P‐*value of 0.05. To identify DMRs unique to the ‘BABA2’ treatment group, overlap was conducted between BABA2 and BABA12 DMRs, with only DMRs unique to BABA2 used for further downstream analysis. To identify genomic features, the ITAG4.1 tomato reference genome was used. Repeat Modeler was used to identify repeats (Hosmani *et al*., 2019). The R package *GenomicRanges* (v.1.46.1) (Lawrence *et al*., 2013) was used to assess overlap between DMRs and genomic features.

### GO enrichment analysis

Gene Ontology (GO) enrichment analysis was performed on DEGs and genes overlapping DMRs (±2 kb). Analysis was performed by using the online tool ShinyGO (Ge *et al*., 2019) with Fisher's exact test, false discovery rate (FDR) correction and a *P‐*value cut‐off of 0.05.

### Grafting

To assess whether BABA‐IR is transmissible through graft junctions, 2‐wk‐old Micro Tom plants were treated to a final concentration of 0.5 mM BABA via soil drench treatment or with water. Three days after soil treatments, grafting was conducted on seedlings using the cleft method (Lee, 1994). The following graft combinations were used with a minimum of 4 biological replicates per treatment: BABA graft (BG) BABA‐treated root stock + BABA‐treated scion; Hetero graft (HG) BABA‐treated root stock + water‐treated scion; and water graft (WG) water‐treated root stock + water‐treated scion.

### Mass spectrometry quantification of BABA residues

Quantification of BABA was performed as described (Thevenet *et al*., 2017) with some modifications. Briefly, ground, freeze‐dried material from tomato fruit was extracted using 500 μl of extraction buffer containing ethanol at 80% and supplemented with internal standard BABA‐d3 to a final concentration of 50 parts per billion (ppb). Fruit material was mixed with the extraction buffer using a mixer mill until homogeneous solutions were obtained. Samples were centrifuged and the supernatant was placed in a clean tube, filtered through a 0.22 μm regenerated cellulose filter (Phenomenex, Torrance, CA, USA) and diluted three times. An aliquot of 5 μl of each extracted sample was injected into a Xevo TQS instrument (Waters Micromass, Manchester, UK) with a T‐wave device and coupled to a triple quadrupole mass spectrometer. For BABA quantification, a Kinetex 1.7 μm HILIC 100 Å LC column 100 × 2.1 mm (Phenomenex) was used.

### Library preparation and sequencing sRNA data

sRNA sequencing analysis was conducted on RNA extracted from scion leaf tissue from BG, HG and WG plants 6 wk postgrafting. Library preparation and sequencing were conducted by Novogene using NovaSeq SE50. Twenty million paired reads were generated per sample. A total of 302 217 035 clean reads were generated across 12 samples, with an average of 25 184 752 clean reads per sample. An average of 97.5% of nucleotides per sample had a Phred quality score of > 30. All sequencing information is summarised in Table S1.

### Analysis of sRNAs

Quality of data was assessed using Fast qc. Samples were trimmed using Trimmomatic (v.0.39) to remove adapter sequences and obtain reads between 16 and 30 bp in length (Bolger *et al*., 2014). Trimmed reads were uniquely mapped to the reference genome using Shortstack (v.3.8.5) (Axtell, 2013). Clusters were identified as 20–24 nt in size using the *mobileRNA* package (https://github.com/KJeynesCupper/mobileRNA). For each replicate, the consensus cluster size was identified and nondicer‐derived clusters were excluded from the analysis. Differentially expressed clusters were identified between HG and WG, BG and WG and HG and BG using the deseq2 method. Location of DE clusters was found using *GenomicRanges* (v.1.46.1) (Lawrence *et al*., 2013) by overlapping clusters (±1 kb) with known genomic features using a log threshold change of 1 or −1 for up and downregulated, respectively; a FDR threshold of 0.05 was applied to control significance.

## Results

### BABA‐induced resistance occurs in tomato if plants are treated at an early developmental stage

**Fig. 1:**
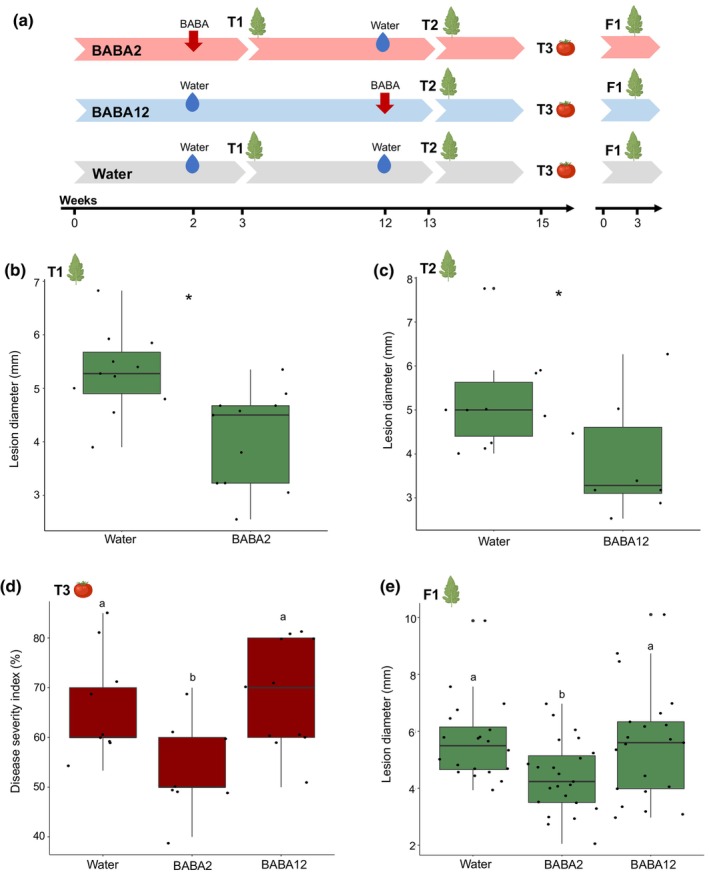
Tomato phenotypic data. (a) Experimental setup. Plants were treated with 0.5 mM β‐aminobutyric acid (BABA) via soil drench at either 2 wk (BABA2) or 12 wk (BABA12). Samples for transcriptome analysis were collected at T1 (1 wk after BABA2 treatment) and at T3 (13 wk and 3 wk after BABA2 and BABA12 treatments, respectively). Samples for DNA methylation analysis were collected at T1 (1 wk after BABA2 treatment), T2 (13 wk and 1 wk after BABA2 and BABA12 treatments, respectively) and at T3 (15 wk and 3 wk after BABA2 and BABA12 treatments, respectively). (b) Lesion diameter in 3‐wk‐old tomato leaves caused by *Botrytis cinerea* at T1 at 3 d postinoculation (dpi). (c) Lesion diameter in 13‐wk‐old tomato leaves caused by *B. cinerea* at T2 at 3 dpi. Asterisks denote significant differences between treatment groups (*t*‐test, *P* < 0.05, *n* = 8–10). (d) Disease severity index in ripe tomato fruit of primed plants, converted from the percentage of lesions in six disease categories at 2 dpi with *B. cinerea* at T3, 15‐ and 3 wk after BABA2 and BABA12 treatments, respectively. (e) Lesion diameter in 3‐wk‐old tomato leaves of F1 progeny caused by *B. cinerea* at 2 dpi. Different letters denote significant differences among treatment groups (one‐way ANOVA, Tukey *post hoc* test, *P* < 0.05, *n* = 8–20). Horizontal lines in boxplots indicate the median, boxes indicate the 75 (top) and 25 (bottom) percentiles, and the length of the box is the interquartile range (IQR), whiskers indicate 1.5 time the IQR above and below the mean and data points indicate each biological replicate.

A time course experiment was designed to investigate the dynamics of BABA‐IR establishment in tomato (Fig. 1a). We used a soil drench approach to prime with BABA the roots of plants at two different developmental stages, either at 2 or 12 wk after germination (hereafter referred to as BABA2 and BABA12, respectively). We analysed plant material at three developmental times, including leaves collected at 3 and 13 wk postgermination (referred to as T1 and T2, respectively) and in detached ripe, red fruit (referred to as T3). Water‐treated plants were used as controls for each of the timepoints considered. We assessed *B. cinerea* disease progression based on the size of lesions appearing after infection of detached leaves and fruit from treated plants and in detached leaves of the following generation. We observed short‐term resistance, as measured in leaves 1 wk after BABA treatment, in leaves from both BABA2 (Fig. 1b) and BABA12 (Fig. 1c) treatment groups. Importantly, we observed long‐lasting resistance in fruit and progenies of treated plants, only when plants were treated at 2 wk old (BABA2). Resistance in fruit and progenies of plants treated later in development (BABA12) did not differ from that observed in water‐treated plants (Fig. 1d,e). Although BABA has been reported to cause phytotoxicity which manifests as growth reduction in young seedlings of commercial tomato cultivars (Luna *et al*., 2016), BABA2 treatment caused no statistically significant reduction in RGR for 7 wk after treatment in Micro tom (Fig. S1).

### Short‐term and long‐term BABA priming of gene expression

**Fig. 2:**
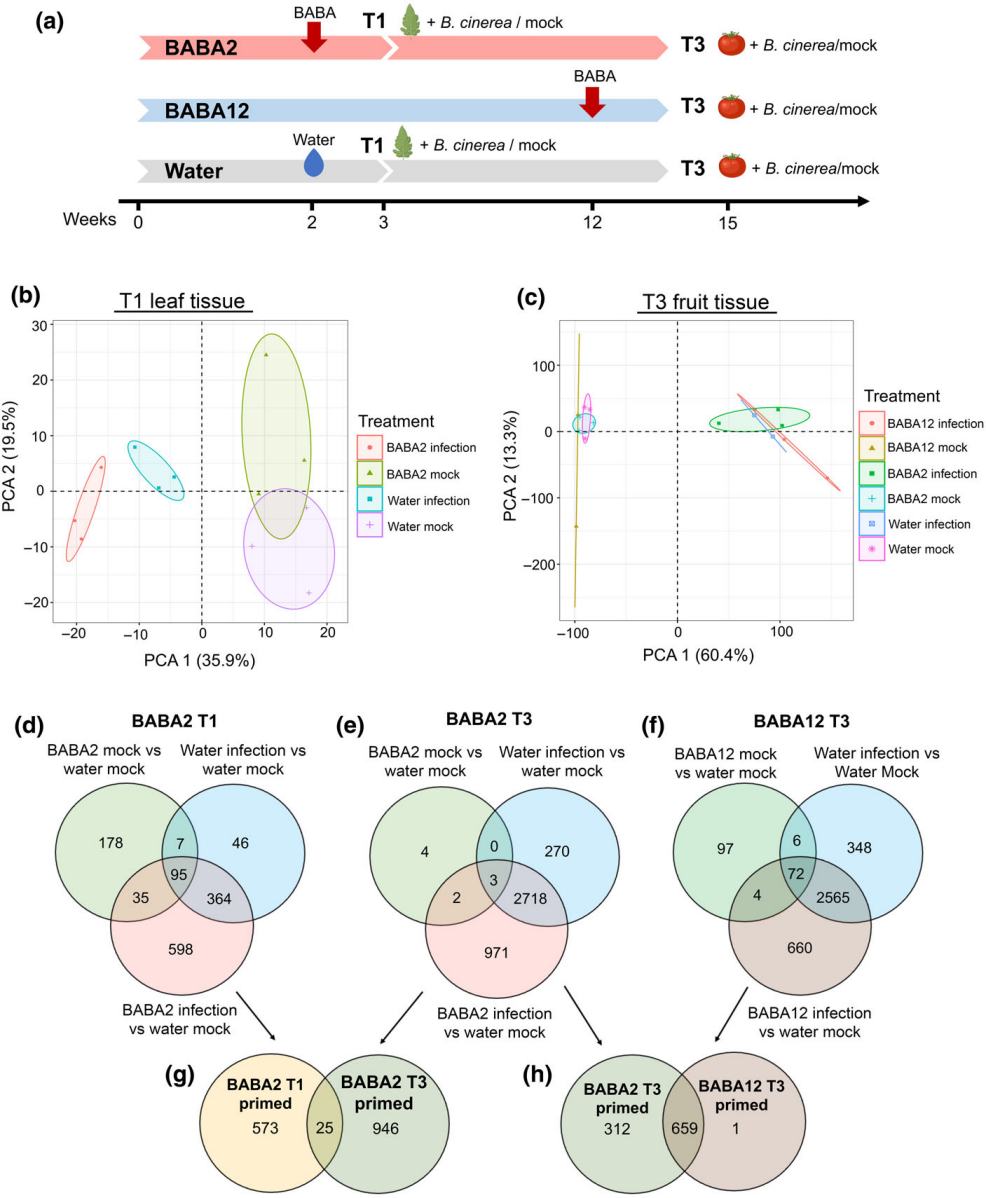
Transcriptome responses of tomato to *Botrytis cinerea* infection. (a) Experimental setup. Plants were treated with 0.5 mM β‐aminobutyric acid (BABA) via soil drench at either 2 wk (BABA2) or 12 wk (BABA12). Samples for transcriptome analysis were collected in *B. cinerea* and mock‐treated leaf tissue (T1) and fruit tissue (T3). Principal component analysis (PCA) of transcriptomic responses to *Botrytis cinerea* infection or mock inoculation in (b) early BABA‐treated (BABA2) and water‐treated 3‐wk‐old tomato leaf tissue at T1 and (c) BABA2‐, water‐ and BABA12‐treated fruit tissue at T3. Venn diagram comparisons between treatment‐specific differentially expressed genes: (d) BABA2 mock vs water mock, water infection vs water mock and BABA2 infection vs water mock at T1, (e) BABA2 mock vs water mock, water infection vs water mock and BABA2 infection vs water mock at T3, and (f) BABA12 mock vs water mock, water infection vs water mock and BABA12 infection vs water mock. (g) BABA2 T1‐primed genes vs BABA2 T3‐primed genes, (h) BABA2 T3‐primed genes vs BABA12 T3‐primed genes.

To test the priming mechanisms activated by BABA, transcriptomic analysis was conducted on *B. cinerea*‐infected tissues. To characterise specific short‐ and long‐term priming, analysis was conducted on infected leaf tissue (T1) and infected fruit tissue (T3), respectively (Fig. 2a). We analysed samples when disease symptoms were first observed at 24 and 48 h after infection in leaves and fruit, respectively. Principal component analysis revealed a clear separation between mock‐inoculated and infected samples in the short‐term response (T1). Furthermore, PC1 also differentiated BABA2 from water‐infected leaves (Fig. 2b). In fruit tissue (T3), the impact of the infection was even more pronounced than in leaves at T1 (Fig. 2c). However, all the infected samples regardless of the BABA treatment (i.e. BABA2 and BABA12), clustered together, indicating that in fruit tissue the infection is the major influence of the transcriptomic response. This result indicates that the BABA treatment has a stronger effect on gene expression in leaves at T1 shortly after its application, than in fruit at T3.

Since long‐lasting resistance in fruit was only conferred by the BABA2 priming treatment, we reasoned that by comparing transcriptional responses to infection between the different priming treatment groups, we could distinguish those primed genes associated with long‐lasting priming from those showing other effects of BABA treatment. To this end, we first identified primed genes from the BABA2 treatment group. Primed genes (represented in the lower sector of the Venn diagrams in Fig. 2d,e), were defined as those differentially expressed in *Botrytis*‐infected tissues of BABA‐treated plants, but which were not also independently differentially expressed following BABA treatment in uninfected tissues or following infection of tissues from water‐treated control plants. Genes primed by BABA2 treatment during infection of leaves at 3 wk (T1) were labelled as ‘BABA2 T1 primed’ and those primed in fruit at 15 wk (T3), as ‘BABA2 T3 primed’. A total of 598 ‘BABA2 T1 primed’ and 971 ‘BABA2 T3 primed’ genes were identified by this approach. Therefore, it appears that BABA treatment of seedlings impacts a greater number of genes over the long term compared with the short term. We also compared the composition of these two sets of primed genes. Only 25 out of a total 1569 genes were shared (Fig. 2g), indicating that both the timing of the expression of priming and tissue type are major drivers of the transcriptomic priming profiles. Following the same approach taken for BABA2, we next identified genes primed during infection of fruit (T3) in the BABA12 treatment group. We found 660 ‘BABA12 T3 primed’ genes (Fig. 2f). However, since BABA12 displayed a susceptible phenotype in fruit tissue (Fig. 1b), it is unlikely that ‘BABA12 T3 primed’ genes include genes directly related to the long‐term resistance phenotype. Finally, we compared T3‐primed genes from BABA2 and BABA12 fruit (Fig. 2h). The majority of DEGs were primed by both BABA2 and BABA12 treatments (Fig. 2h). These genes were enriched in pathways such as ‘response to abiotic stimulus’ and ‘response to oxygen‐containing compound’ (Fig. S2a). We identified 1 gene corresponding to a TIFY 10A‐related gene, that is primed exclusively by the BABA12 treatment (Fig. 2g). Moreover, we identified 312 genes that are primed exclusively by the early BABA2 treatment. These genes were enriched in pathways relating to ‘Metabolic pathways’ and ‘Photosynthesis’ (Fig. S2b). These genes might therefore be linked to long‐term resistance in fruit tissue and were considered further in subsequent analyses.

### DNA methylation changes induced by BABA are dynamic and increase over time

**Fig. 3:**
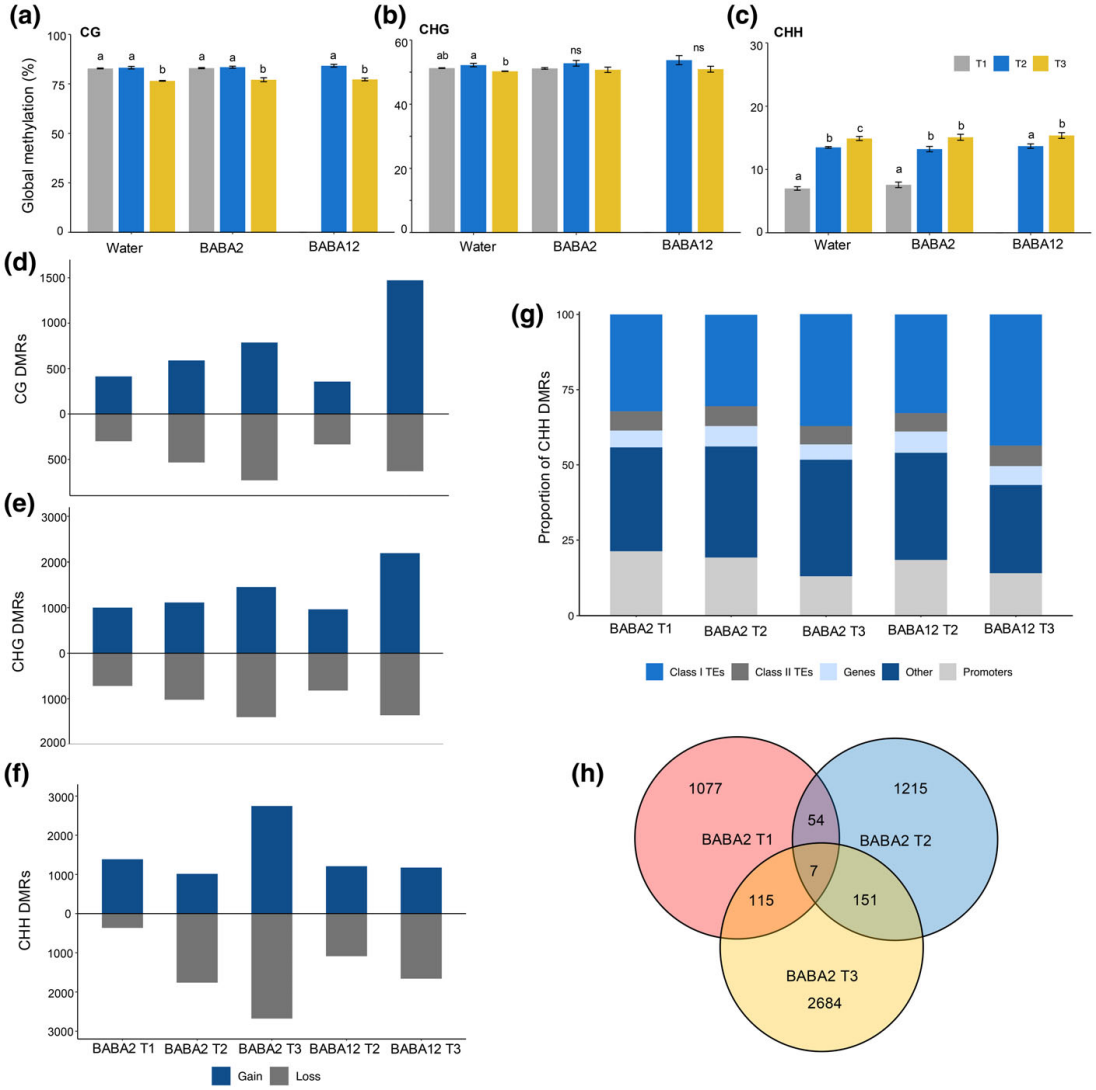
Whole‐genome bisulfite sequencing (WGBS) analysis in tomato. Global methylation level across treatment groups in 3‐wk‐old leaf tissue (T1; grey), 13‐wk‐old leaf tissue (T2; blue) and ripe fruit tissue (T3; yellow) in each cytosine context: (a) CG, (b) CHG and (c) CHH. Error bars represent the standard error of the mean (SEM). Letters indicate statistically significant differences between timepoints (ANOVA or pair‐wise *t*‐test; *P* < 0.05; *n* = 3). ns indicates not significant. A total number of hyper‐differentially methylated regions (DMRs) (blue) and hypo‐DMRs (grey) across samples in each cytosine context: (d) CG, (e) CHG and (f) CHH. (g) Location of DMRs across genomic figures in BABA2 leaf tissue at 3 wk. (h) Venn diagram illustrating correspondence between genes overlapping DMRs unique to BABA2 treated plants at T1 (red) T2 (blue) and T3 (yellow).

To explore whether epigenetic mechanisms are involved in long‐term priming, we investigated changes in DNA methylation following treatments with BABA in each of the samples collected at the different timepoints of our analysis. We conducted whole‐genome bisulfite sequencing (WGBS) to obtain DNA methylation profiles of each sample at single cytosine resolution. No significant response to BABA treatment was seen at the global methylation level when each treated sample was compared with the corresponding water control at the same timepoint. However, we did observe statistically significant differences in global DNA methylation depending on tissue type and developmental stage (Fig. 3a–c). For CG, we observed lower levels of global methylation at T3 (fruit; 77.9%) than at T1 and T2 (leaves; 82.9% and 83.6%, respectively) (Fig. 3a). Reduced methylation in fruit was also observed in the CHG context in water‐treated plants, where we detected a reduction in global DNA methylation at T3 (fruit; 50.3%) compared with T1 and T2 (leaves 51.2% and 52.2%, respectively; Fig. 3b). Moreover, changes associated with the developmental stage were also observed in the CHH context, where global levels increased from 7.3% to 13.5% between T1 and T2 and to 15.4% in T3 in all treatment groups (Fig. 3c). Therefore, we only observe differences between the global methylation patterns in leaves of different developmental stages in the CHH context.

To investigate the presence of specific DNA methylation patterns associated with BABA priming, we identified DMRs from samples treated with BABA and the corresponding water control, for each timepoint. The total numbers of DMRs found in each context are listed in Table 1.

**Table 1:** Total number of 200 base pair differentially methylated regions (DMRs) in each cytosine (C) context (CG, CHG and CHH) in tomato.

Timepoint	Treatment vs water	CG	CHG	CHH
T1	BABA2	714	1720	1751
T2	BABA2	1125	2137	2777
	BABA12	692	1783	2297
T3	BABA2	1518	2858	5425
	BABA12	2104	3552	2836

Generally, the number of DMRs increases over time in all three cytosine contexts. This effect is most dramatic in the CHH context where the total number of DMRs increases over three times from T1 to T3 and 2 times from T2 to T3 in BABA2 plants (Table 1). By contrast, there was only a small increase in CHH DMRs between T2 and T3 for BABA12. There was a more noticeable increase in the numbers of CG and CHG DMRs in BABA12, which was not as pronounced in BABA2. Collectively, these results suggest that DNA methylation in tomato is primarily affected by the developmental stage, displaying increased variation in the CHH context and, less markedly, in the CG context. These results also indicate that the most prominent effect of BABA treatment occurs several weeks after the treatments, with the maximum difference measured in fruits, and many new DMRs arising weeks after BABA2 treatment.

Major differences were identified in the gain and loss of the methylation status of DMRs depending on the timepoint of BABA application, particularly in the CHH context (Fig. 3d–f). At T1 BABA2, DMRs were more often hyper‐ than hypomethylated, with the greatest effect observed in the CHH methylation context, where four times more DMRs were hypermethylated. This gain of CHH DMRs was not maintained in the later timepoints: CHH DMRs at T2 showed greater loss and at T3 gain and loss were equal (Fig. 3f). By contrast, BABA12 did not lead to the same pattern of DMR imbalance in any cytosine context and gain/loss ratios were maintained at T2 (Fig. 3d–f). At T3 however, there was a gain of methylation in CG and CHG contexts, and a loss in CHH methylation. We observed that for all samples analysed, DMRs were primarily concentrated on Class I TEs for all cytosine contexts (Fig. 3g), but also overlap a portion of genes and/or their promoters. Therefore, to identify differentially methylated genes (DMGs) that potentially contribute to priming, we isolated DMGs unique to the BABA2 phenotype. We identified 1253 DMGs unique to BABA2 at T1, 1427 at T2 and 2957 at T3. Overlap of these DMGs revealed that only 54 were present in both T1 and T2, 115 in T1 and T3, 151 in T2 and T3. Only seven DMGs were present at all three timepoints (Fig. 3h). This indicates that early DMRs generated by BABA soil drench treatment at 2 wk are generally not maintained in older leaves or fruit tissue.

### Genes associated with priming are not frequently methylated

To test whether transcriptional responses associated with BABA treatments are epigenetically regulated, DEGs resulting from the BABA treatment alone (i.e. BABA mock – without infection; Fig. 2d–f) and ‘BABA2 T1 primed’, ‘BABA2 T3 primed’ and ‘BABA12 T3 primed’ genes (Fig. 2g,h) were compared with DMGs. Venn diagram analysis revealed that very few genes associated with a BABA transcriptional response were also differentially methylated (Fig. S3a–c). Whereas at T1, only 25 out of 573 ‘BABA2 T1 primed’ genes were also differentially methylated in any cytosine context (Table S2), no DEGs responding to early BABA treatment without infection were differentially methylated at T3 and only 16 genes were both ‘BABA2 T3 primed’ and differentially methylated (Fig. S3b; Table S2). Gene Ontology term enrichment analysis was conducted on the full nonredundant lists of DEGs and DMGs contained in the Venn diagrams in Fig. S3(a–c), which identified very little similarity between the biological processes impacted by BABA2 at T1 (Fig. S3d), BABA2 at T3 (Fig. S3e) and BABA12 at T3 (Fig. S3f). Specifically, BABA2 T1 were enriched in ‘Regulation of systemic acquired resistance’ and ‘Photosynthesis, light harvesting’ (Fig. S3d). BABA2 T3 genes displayed enrichment in ‘Endosperm development’ and ‘rRNA (metabolic) processing’ (Fig. S3e). Finally, BABA12 T3 genes were enriched in ‘Hexose catabolic processes’ and ‘Mitochondrial cytochrome c oxidase activity’ (Fig. S3f). This provides further evidence to support the observation that different pathways are associated with short‐term and long‐term priming mechanisms generated by BABA and that the timing of application is crucial for the priming of specific pathways that result in enhanced resistance in fruit.

### Long‐lasting resistance is transmitted from BABA‐primed rootstocks to scions across a graft junction

**Fig. 4:**
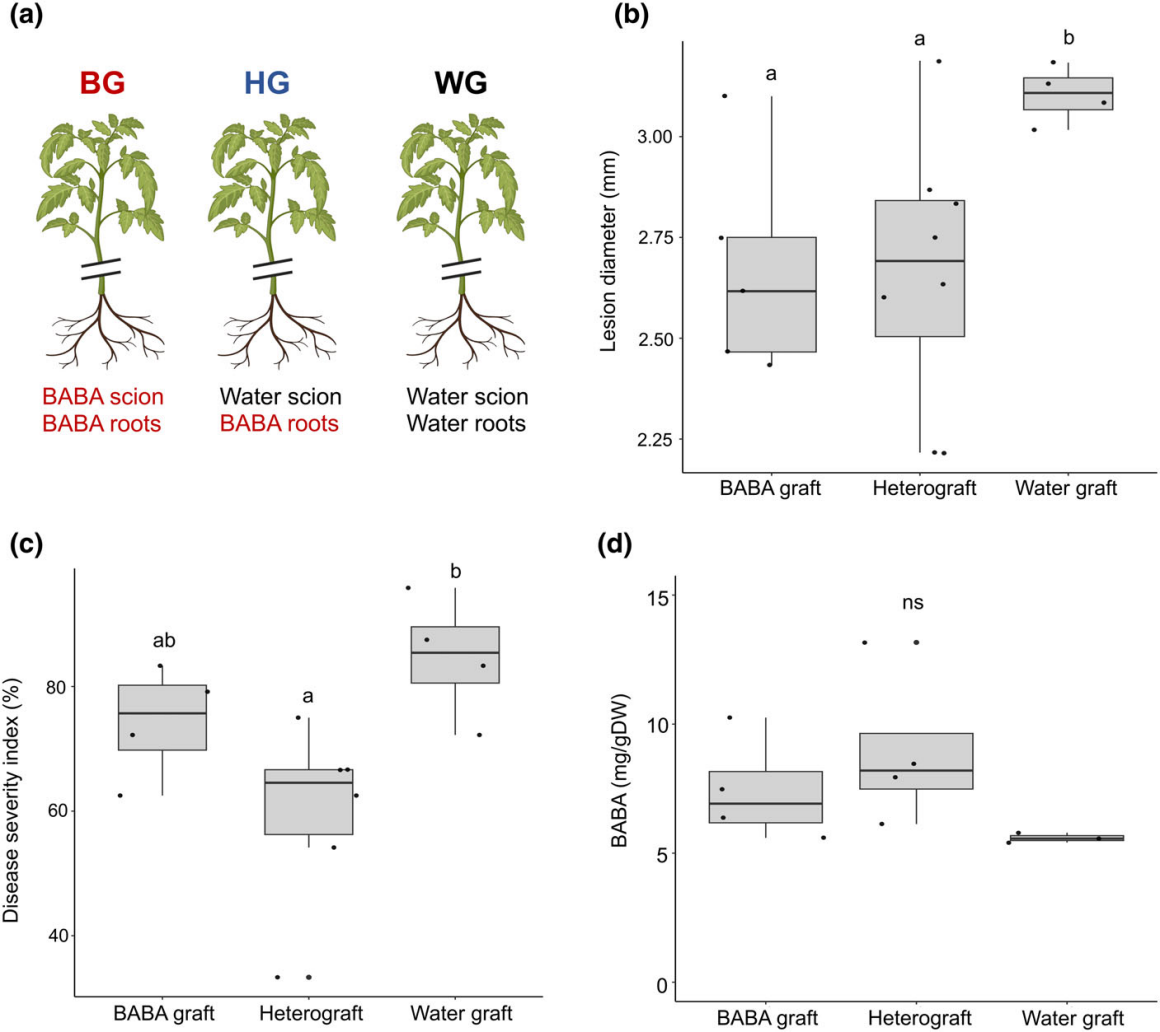
Grafting on β‐aminobutyric acid (BABA) tomato rootstocks. (a) Experimental setup for grafting: from left to right BABA graft (BG) consisting of BABA scion and BABA‐treated roots scion, heterograft (HG) consisting of water scion and BABA roots and water graft (WG) consisting of water scion and water roots. (b) Lesion diameter in tomato leaves infected with *Botrytis cinerea* at 3 d postinoculation (dpi) and (c) disease severity index in ripe tomato fruit converted from the percentage of lesions in six disease categories at 6 dpi with *B. cinerea*. Different letters denote significant differences among treatment groups (one‐way ANOVA, least significant differences *post hoc* test, *P* < 0.05, *n* = 3–8). (d) Accumulation of BABA in harvested red fruit of grafted tomato plants (*n* = 3–5). ns indicates not significant. Horizontal lines in boxplots indicate the median, boxes indicate the 75 (top) and 25 (bottom) percentiles, and the length of the box is the interquartile range (IQR), whiskers indicate 1.5 time the IQR above and below the mean, and data points indicate each biological replicate.

We observed that BABA2 treatment triggers long‐lasting IR in tissue which develops after the treatment, and that DMRs specifically associated with BABA2 emerge weeks after the treatment. These observations suggest that long‐lasting resistance after BABA treatment may be maintained via a systemic signal. To test this hypothesis directly, grafting experiments were conducted to determine whether BABA‐treated tissues could transmit resistance to untreated tissues. We grafted naive scions obtained from plants treated with water onto BABA2‐treated rootstocks (hetero graft; HG). As controls, we generated homografts made using both rootstocks and scions obtained from plants treated with BABA (BABA graft; BG) or water‐treated (WG) (Fig. 4a). Resistance against *B. cinerea* was tested in scion leaf tissue 6 wk after grafting (corresponding to 6 wk and 3 d after BABA treatment) and in ripe red fruit tissue. Remarkably, we observed that scion and fruit from HGs displayed the same level of resistance as observed in BGs, with significantly reduced disease symptoms compared with the WG condition (Fig. 4b,c). Mass spectrometry analysis was conducted to determine whether residual traces of BABA in fruit of BABA‐treated rootstocks may be driving long‐lasting resistance observed. BABA was detected in all fruit, including those treated with water. While BABA‐treated rootstock plants displayed higher levels (Fig. 4d), this was not statistically significant (*P‐*value = 0.207). Therefore, our results indicate that BABA‐IR is transmissible to untreated scions by grafting and this resistance phenotype is independent of BABA residues from the rootstock treatment.

Considering that no statistically significant differences were seen in the quantity of BABA in resistant grafted fruit, and that the presence of BABA is insufficient for postharvest resistance, alternative determinant factors were explored. Given that the greatest changes in DNA methylation in our resistance phenotype were found in the CHH context and that this type of methylation is known to be mediated by RdDM, we tested whether the resistance phenotype in HGs is also associated with a change in accumulation of sRNAs. Scion leaf tissue was harvested 6 wk after grafting and sRNA sequencing was conducted (Fig. S4). Differentially expressed (DE) clusters were identified by comparing BG and HG with the WG clusters. In all cases, most of DE clusters were sRNAs 24 nt in size – (HG vs WG: 47%, HG vs BG: 92%, BG vs WG: 85%). We observed a much larger number of DE clusters in the BG plants (17 323 upregulated and 100 298 downregulated) than the HG plants (10 150 upregulated and 38 757 downregulated). By contrast, HG vs BG contained 14 790 upregulated DE clusters and 108 516 downregulated DE clusters. This result indicates that the HG scions maintained an intermediate sRNA profile between WG and BG plants but are more similar to the former condition. This suggests that BABA treatment induced both graft‐transmissible and nontransmissible effects on sRNA accumulation.

**Fig. 5:**
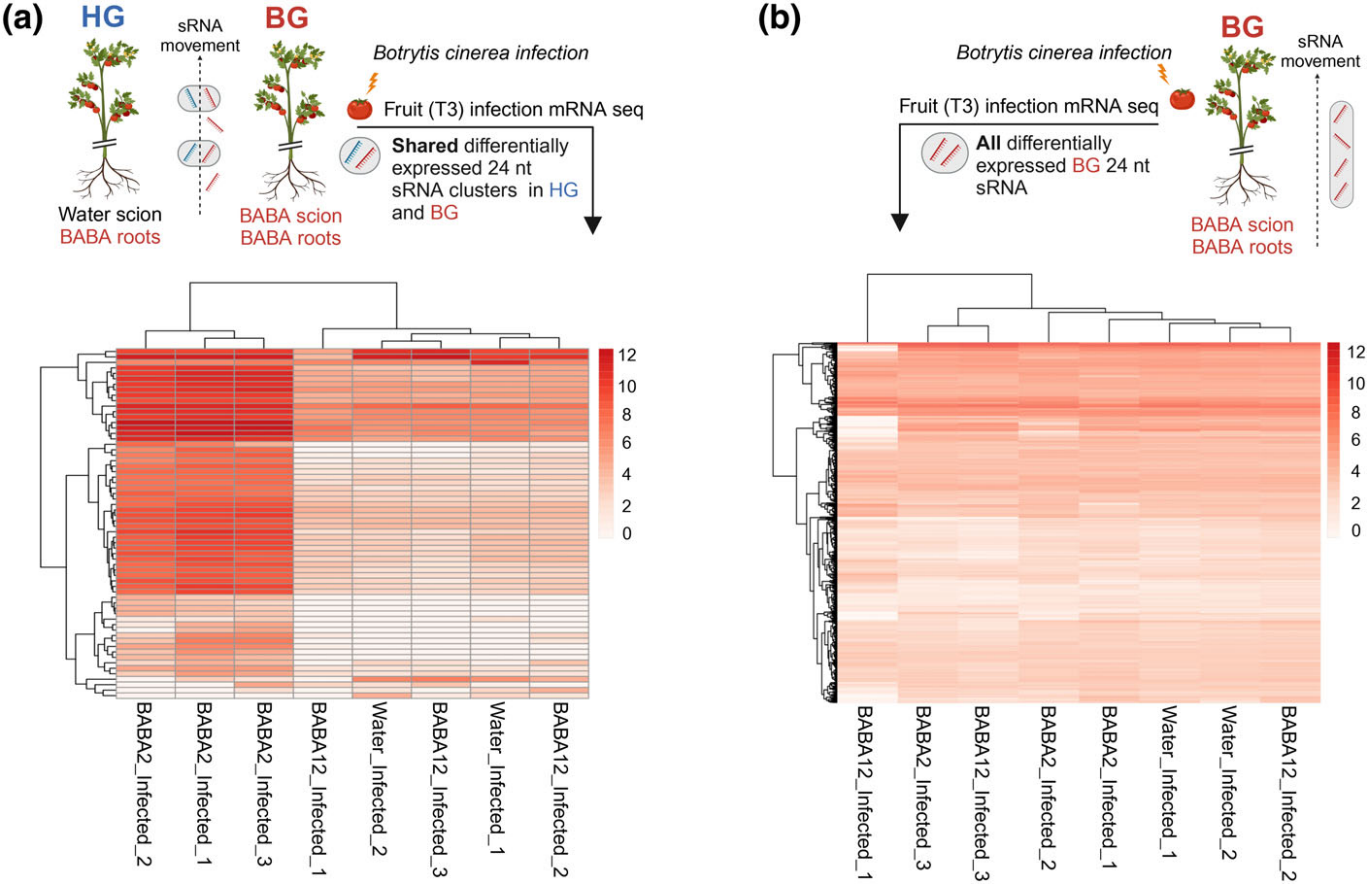
Isolation of mobile small RNAs (sRNAs) in tomato. Heatmap displaying normalised fragments per kilobase of transcript per million mapped reads (FPKM) value of genes containing (a) upregulated differentially expressed 24 nucleotides (nt) sRNA clusters conserved to HG (heterograft – blue) and BG (β‐aminobutyric acid (BABA) graft – red) phenotype captured in grey horizontal oval circles. FPKM values display expression level in fruit tissue of BABA2‐infected, water‐infected and BABA12 *Botrytis cinerea*‐infected plants. (b) Upregulated differentially expressed 24 nt sRNA clusters in BG phenotype (red) captured in a grey vertical oval circle. FPKM values display expression level in fruit tissue of BABA2‐infected, water‐infected and BABA12 *B. cinerea*‐infected plants. Figure was created in BioRender (BioRender.com/i39k866).

To identify BABA‐induced effects more directly linked to long‐term resistance, we identified differentially expressed 24 nt clusters present in both resistant phenotypes (HG and BG) compared with control (WG), producing a total list of 6010 clusters (Table S3). From these, we identified 135 and 119 genes overlapping upregulated and downregulated 24 nt size clusters, respectively. To assess whether these sRNAs were associated with priming responses, the expression levels during *B. cinerea* infection of these overlapping genes were assessed by comparing their fragments per kilobase million (FPKM) values across all treatment groups for infections at T1 (Fig. S5) and T3 (Fig. 5a).

In the 135 genes coinciding with upregulated sRNA clusters, we observed a clear trend of enhanced overexpression in response to infection in the BABA2 T3, compared with all other conditions (Figs 5a, S5a). For these genes, statistically significant enrichment was observed in the following biological process: ‘COPII‐coated vesicle budding’ (103.4‐fold enrichment, FDR *P‐*value 0.025), ‘Aspartate family amino acid biosynthetic processes’ (74.4‐fold enrichment, FDR *P‐*value 0.025), ‘Vesicle budding from membrane’ (71.6‐fold enrichment, FDR *P‐*value 0.025) and ‘Aspartate family amino acid metabolic processes’ (51.7‐fold enrichment 0.029 *P‐*value FDR). Notably, such enhanced response to infection seen in BABA2 T3 is largely specific for these 135 genes, and it is not observed when all genes overlapping upregulated 24 nt clusters unique to the BG condition are considered (2279 genes) (Figs 5b, S5b). Similarly, this effect was not observed for the BABA2 T3 119 genes associated with downregulated clusters (Fig. S5c), or in the BABA2 T1 condition for genes associated with either upregulated or downregulated clusters (Fig. S5e,f). Therefore, our results suggest that mobile sRNAs accumulating in resistant naive tissue grafted on BABA‐primed rootstock are specifically associated with a set of genes with an enhanced response to infection in tomato fruit expressing long‐lasting IR.

## Discussion

Here, we have demonstrated that plant age is a critical factor for the generation and maintenance of long‐lasting IR through chemical priming of defence. We have identified that BABA treatment of 2‐wk‐old tomato seedlings (BABA2) leads to long‐lasting postharvest and heritable resistance, and that this resistance phenotype is not observed if BABA treatment is conducted in 12‐wk‐old plants (BABA12) (Fig. 1b). Moreover, transcriptomic analysis demonstrated that the resistance observed in BABA2‐treated plants is associated with priming of gene expression, which was specific to the tissue and the timepoint analysed. These results suggest that young plants respond to BABA treatments differently than older plants. It is well known that due to the sessile nature of plants, lasting ‘memories’ can be created over the plant's lifetime to facilitate survival and adaptation to environmental pressures (Bruce *et al*., 2007). This memory formation and maintenance has been demonstrated to occur via changes to epigenetic marks (Hannan Parker *et al*., 2022). Moreover, significant epigenetic modifications in chromatin structure and DNA methylation are known to occur during development (Lucibelli *et al*., 2022). For example, 2‐d‐old Arabidopsis leaves have a heterochromatin content of 6% which increases to 14% at 2 wk, thus increasing chromatin packing and impacting transcriptional activity (Mathieu *et al*., 2003; Exner & Hennig, 2008; Hemenway & Gehring, 2023). We used this understanding as a basis to investigate whether the postharvest resistance observed in seedlings could be due to development‐dependent differences in epigenetic profiles. Evidence of a dynamic epigenetic landscape throughout their lifetime is consistent with the idea that young plants could display greater epigenetic plasticity, thus facilitating robust memory formation. We, therefore, suggest that BABA seedling treatment leads to early foundational changes within the epigenome, facilitated by the young plant's enhanced plasticity. In addition, these changes might result in pivotal effects in terms of capacity for subsequent epigenetic reprogramming and consequently, gene expression.

Interestingly, our DNA methylation analysis displayed negligible changes in the global methylation levels of DNA methylation following BABA treatment at 2 or 12 wk (Fig. 3a–c), while similar previous experiments have found that BABA dramatically induces global DNA hypomethylation in the tomato variety Money‐Maker, particularly in the CHH context (Catoni *et al*., 2022). This contrasting observation within the same plant species and priming elicitor could be due to the known differences among tomato varieties in perception of BABA (Luna *et al*., 2014) and in the dynamic resource reallocation from growth to defence, which manifests mainly as a growth reduction (van Hulten *et al*., 2006; Luna *et al*., 2016). We, therefore, speculate that the global changes in methylation levels in Money‐Maker are associated with BABA‐induced stress upon perception of the elicitor.

Despite the absence of global methylation changes found between treatments in our experiments (Fig. 3a–c), DMR analysis unravelled clear differences between short‐term and long‐term priming responses. Specifically, we observed that early treated plants display a unique pattern of CHH hyper‐DMRs and a higher number of DMRs months after the initial priming treatment, with BABA2 fruit having nearly double the number of CHH DMRs than BABA12 (Fig. 3f). These observations could be directly associated to IR, as CHH methylation has been described to play a relevant role in response to stimuli and stress (López *et al*., 2011) and has become recognised as an epigenetic modification marking the maintenance and expression of long‐lasting priming (Wang *et al*., 2021; Catoni *et al*., 2022). Nonetheless, DMRs created by the BABA2 treatment are not maintained throughout the life of the plant. This supports the hypothesis that while BABA treatment has a pivotal influence on the future DNA methylation landscape, the early methylation response to BABA may not be directly associated with the establishment of long‐term IR. Moreover, CHH methylation levels in leaf tissue double over 10 wk from 7.3% to 13.5% (Fig. 3c), indicating a highly dynamic profile of this epigenetic mark in tomato development. This agrees with various studies that have demonstrated that global methylation levels increase with chronological age in many plant species (Valledor *et al*., 2010; Dubrovina & Kiselev, 2016). Taking this observation into consideration, we can argue that the lower global CHH level in young seedlings is associated with a greater plasticity in young plants and facilitates a strong priming imprinting following BABA treatment.

The observation that DMRs associated with BABA2 are not maintained from seedlings to fruiting (Fig. 3h) further supports the hypothesis on the importance of foundational changes at early stages of development and tissues that experience early BABA exposure. Considering that tissues present early during development are influencing responses in fruit that were not present at the time of BABA2 treatment, this brings the question of whether the long‐lasting resistance is due to a systemic signal. We, therefore, combined the use of grafting and BABA soil drench treatments. Our experiments demonstrate that long‐lasting resistance is transmissible across graft junctions to untreated naive scions (Fig. 4b,c). The absence of long‐term resistance observed in BABA12 plants excludes that this primed state is due to residual BABA transported to and accumulated in fruit tissue (Wilkinson *et al*., 2018). Importantly, no statistically significant differences were observed between graft plant treatment conditions, with relatively large quantities (5 mg g^−1^ DW) detected even in fruit of more susceptible water‐treated plants (Fig. 4d).

Our efforts then focused on the characterisation of this mobile signal by investigating the accumulation of sRNAs, which are known to be mobile and able to transmit traits across a graft junction (Jeynes‐Cupper & Catoni, 2023). Previous grafting experiments performed with transgenic plants have demonstrated that 24 nt sRNAs lead to epigenetic changes in recipient scion tissue (Molnar *et al*., 2010). Importantly, the RdDM pathway has been frequently associated with defence against biotic stresses and with the expression and maintenance of long‐lasting priming (López *et al*., 2011; López Sánchez *et al*., 2016). Therefore, one could speculate that the RdDM pathway plays a role in the establishment and maintenance of long‐lasting priming in tomato by impacting mobile sRNAs that alter methylation in naive tissue.

To identify sRNAs potentially driving the resistance observed in our experiments, sRNA sequencing was conducted on leaf tissue from grafted plants. Interestingly, while BABA‐treated plants (BG) display a change in the accumulation of many sRNA clusters, only a small portion are maintained in grafted scions (HG) and are therefore associated with long‐lasting resistance. This result suggests that most BABA‐induced effects on sRNA accumulation are not involved in graft‐transmissible IR. Conserved differentially expressed 24 nt sRNA clusters were identified from our resistant plants (BG and HG). The 135 genes associated with upregulated clusters displayed a clear pattern of enhanced expression during fruit infections in resistant BABA2 plants (Fig. 5a). This is a relatively unexpected result, as the production of sRNAs are commonly associated with gene silencing through RdDM (Molnar *et al*., 2010). However, enhanced expression of these cluster‐associated genes was not observed in BABA12 or water plants. Moreover, the same expression pattern was not observed in leaf tissue or across our downregulated cluster‐associated genes, (Fig. S5c–f). Whereas these phenotypes could be due to the dynamic nature of transcriptomic and epigenomic responses during both priming (Meijer *et al*., 2023) and pathogen infection (De Vega *et al*., 2021), the regulation of transposable elements (TE) (Wilkinson *et al*., 2023) and the limitations of collecting single timepoints (Guan *et al*., 2020), our results suggest that early BABA treatment generates a systemic signal that leads to dynamic changes in the priming response in the fruit.

Among the genes that we found functionally associated with sRNA clusters and priming in our experiment, significant enrichment was observed in pathways including ‘COPII‐coated vesicle budding’ and ‘Aspartate family amino acid biosynthetic process’. These pathways were not enriched during any of our initial omics analyses (Figs S2, S3), likely due to the reasons outlined above regarding the differences tissue type and timepoints. Nevertheless, this specificity demonstrates that there is potential to identify novel mechanisms of immune priming by considering whole‐plant, far‐reaching responses. From the identified pathways, the ‘Aspartate family amino acid biosynthetic process’ is particularly intriguing, since priming by BABA in Arabidopsis has been directly associated with aspartic acid‐related processes, following identification of the BABA receptor as the aspartyl‐tRNA synthetase IBI1 (Luna *et al*., 2014). In addition, an asparagine synthetase was also found in these T3‐primed genes, providing further evidence of a role of amino acid processes related to aspartic acid. Interestingly, this asparagine synthetase has been shown to be important in defence against microbial pathogens and is mainly found in root tissue (Olea *et al*., 2004; Hwang *et al*., 2011), where BABA is applied and taken up.

Our experiments were part of an approach to identify epigenetic markers to develop protection strategies against *B. cinerea* in tomato. The results have the potential to impact the horticulture industry, as they guide it towards priming applications and imprinting of resistance phenotypes at early stages of cultivation, thereby reducing associated commercial costs. Moreover, this work has led to the discovery that long‐lasting BABA‐IR is transmissible through graft junctions, and we have identified a group of sRNAs with a potential role in DNA methylation‐dependent maintenance of long‐lasting priming. Future research will focus on understanding the specific mechanisms by which sRNA mediates resistance through changes in DNA methylation, transcription and a potential role for TEs. Considering the relevance of grafting to the horticulture industry due to enhanced plant vigour (Cerruti *et al*., 2021; Jeynes‐Cupper & Catoni, 2023), our results could be exploited in commercial settings, for both enhanced growth and protection against diseases pre‐ and postharvest.

## Competing interests

None declared.

## Author contributions

EL, MC and MRR conceived the study and obtained core funding. EL, KS and MC designed the experimental work pipeline. KS and LM conducted experiments and gathered data. VP conducted the mass spectrometry experiments. EL, KS, MC and MRR designed the data analysis pipeline. KS performed data analysis with the guidance of KJ‐C for the analysis of the sRNA data. KS and EL performed statistical analyses and data interpretation with the support of MC, MRR and KJ‐C. KS and EL drafted the first version of the article. KS, EL, MC and MRR wrote the submitted version of the article. All authors provided input in the final submitted version.

## Supporting information


**Fig. S1** Relative growth rate of water and BABA‐treated plants.
**Fig. S2** Pathway enrichments of transcriptome analysis.
**Fig. S3** Transcription and DNA methylation overlap and pathway enrichment.
**Fig. S4** Distribution of length of sequenced sRNA.
**Fig. S5** Heatmaps of expression of the sRNA‐associated genes.

**Table S1** Summary of sequencing datasets.
**Table S2** List of genes transcriptionally primed and differentially methylated.
**Table S3** List of sRNA mobile clusters.
Please note: Wiley is not responsible for the content or functionality of any Supporting Information supplied by the authors. Any queries (other than missing material) should be directed to the *New Phytologist* Central Office.

## Data Availability

Sequencing data have been deposited in Gene Expression Omnibus under the accessions GSE273942 (WGBS), GSE273944 (RNAseq) and GSE273943 (sRNAseq).
