# Supplementary material for: Developmentally regulated generation of a systemic signal for long‐lasting defence priming in tomato
Source: New Phytol. 2024 Nov 19;245(3):1145–57. doi: 10.1111/nph.20288 (PMC11711926; doi:10.1111/nph.20288)
Supplement: Supplementary file 1 — Fig. S1 Relative growth rate of water and BABA‐treated plants. Fig. S2 Pathway enrichments of transcriptome analysis. Fig. S3 Transcription and DNA methylation overlap and pathway enrichment. Fig. S4 Distribution of length of sequenced sRNA. Fig. S5 Heatmaps of expression of the sRNA‐associated genes. [file NPH-245-1145-s001.pdf]

New Phytologist

**_New Phytologist_ Supporting Information**

Article title: Developmentally regulated generation of a systemic signal for long-lasting defence priming in tomato
Authors: Katie Stevens, Michael R Roberts, Katie Jeynes-Cupper, Lamya Majeed, Victoria Pastor, Marco Catoni and Estrella Luna
Article acceptance date: 04 November 2024

The following Supporting Information is available for this article:

**Fig. S1** Relative growth rate of water and BABA-treated plants

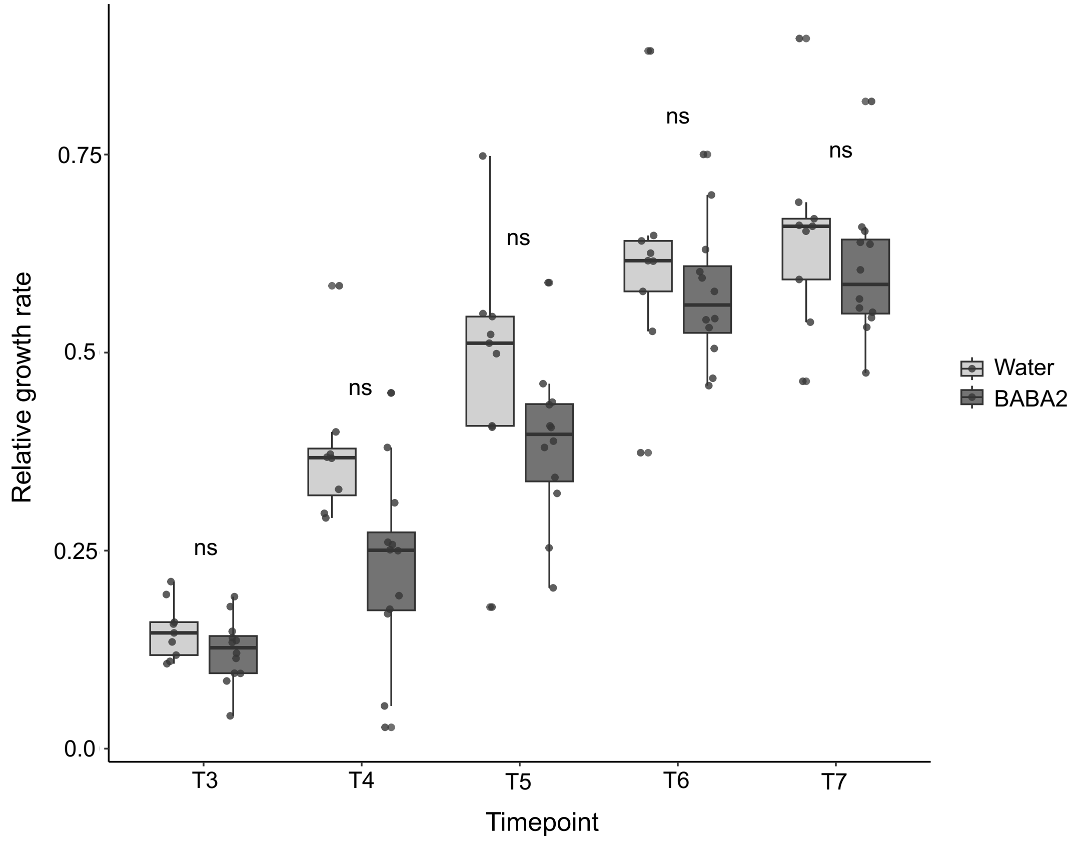


**Fig. S1** Relative growth rate. Average relative growth rates (in cm per cm per week) of plants between 2 and 3, 4, 5, 6, and 7 weeks after 0.5 mM BABA treatment. ns denotes no significant differences among treatment groups (Student's t-test, $p < 0.05$; n = 8-10). Horizontal lines in

boxplots indicate the median, boxes indicate the 75 (top) and 25 (bottom) percentiles and the length of the box is the interquartile range (IQR), whiskers indicate 1.5 time the IQR above and below the mean and data points indicate each biological replicate.

**Fig. S2** Pathway enrichments of transcriptome analysis

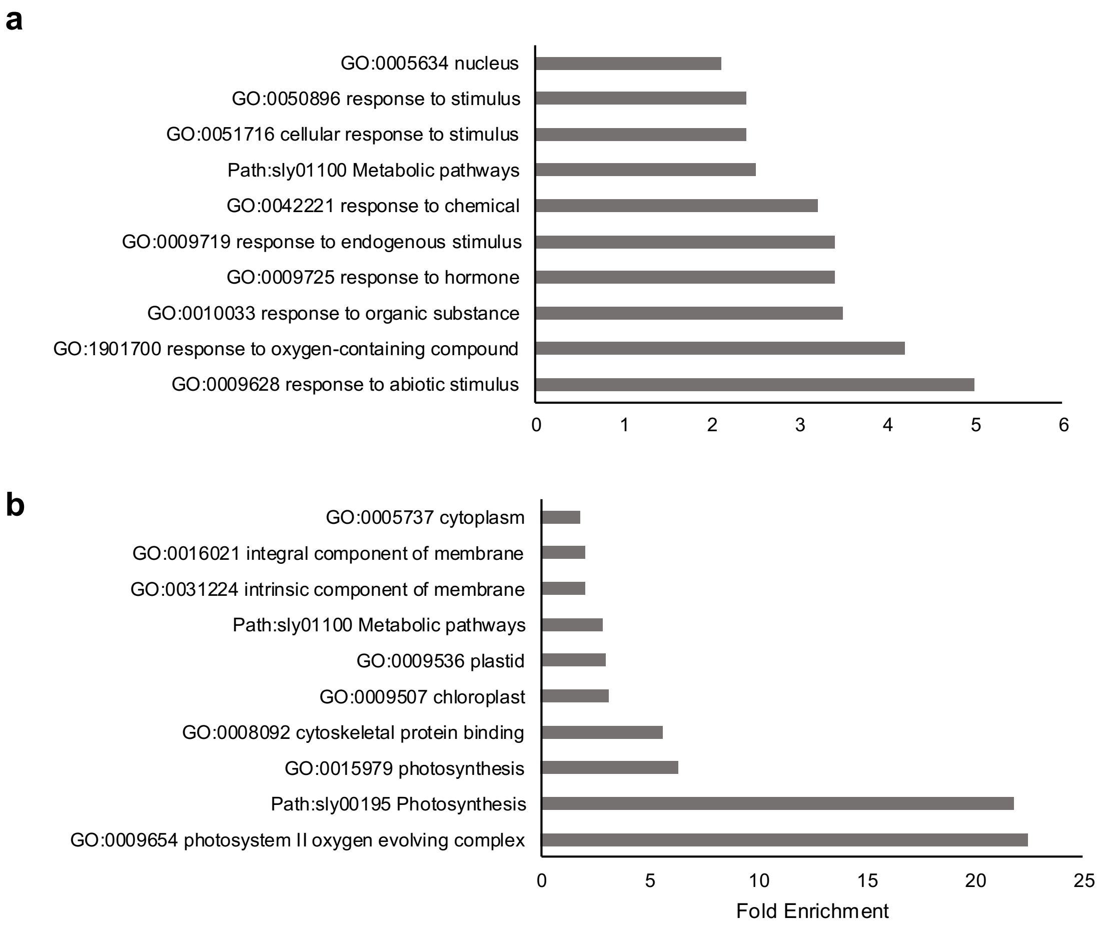


**Figure S2:** Pathway enrichment analysis. a) Gene ontology (GO) term enrichment of 659 genes conserved in BABA2 T3 primed and BABA12 T3 primed, b) GO term enrichment of 312 unique BABA2 T3 primed genes.

## Figure S3: Transcription and DNA methylation overlap and pathway enrichment

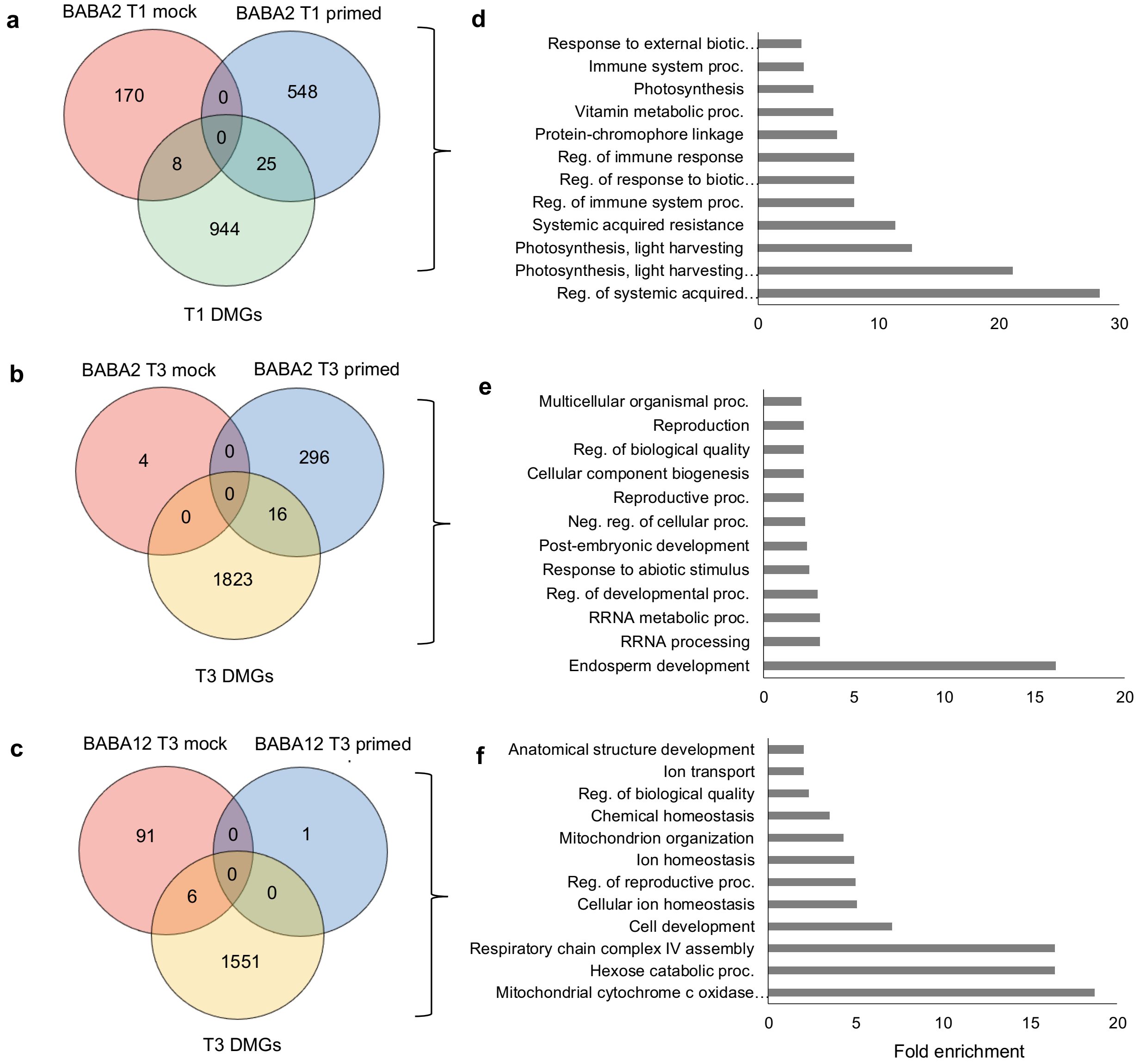


**Fig S3: Integrated transcriptome and methylome analysis.** Overlap of all BABA differentially expressed genes (DEGs) and genes containing differentially methylated regions (DMRs), and their enrichment. a) BABA2 T1 genes (red representing BABA2 mock DEGs, blue representing BABA2 T1 primed DEGs, green representing T1 BABA2 DMGs), b) BABA2 T3 genes (red representing BABA2 mock DEGs, blue representing BABA2 T3 primed DEGs , yellow representing T3 BABA2 DMGs), c) BABA12 T3 genes (red representing BABA12 mock DEGs, blue representing BABA12 T3 primed DEGs, yellow representing T3 BABA12 DMGs), d) Gene ontology (GO) term enrichment of all BABA2 T1 genes, e) GO term enrichment of all BABA2 T3

genes, f) GO term enrichment of all BABA12 T3 genes.

**Figure S4: Distribution of length of sequenced sRNA**

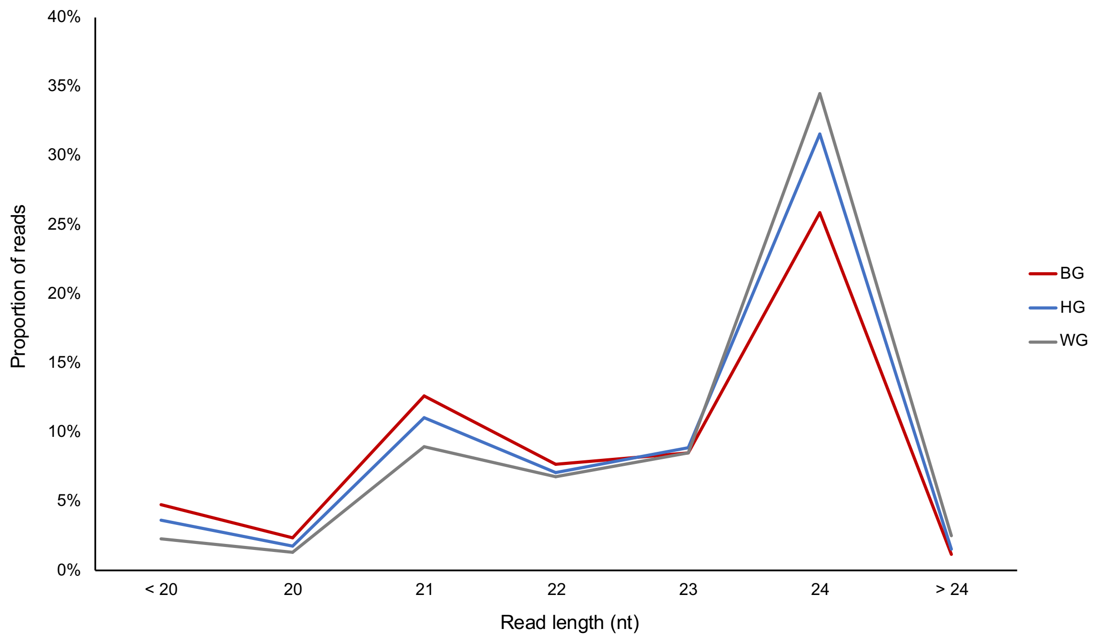


Fig S4: sRNA sequencing sizes. Length distribution of sRNA reads in grafting treatment groups BABA graft (BG, red), heterograft (HG, blue) and water graft (WG, grey).

**Figure S5: Heatmaps of expression of the sRNA associated genes**

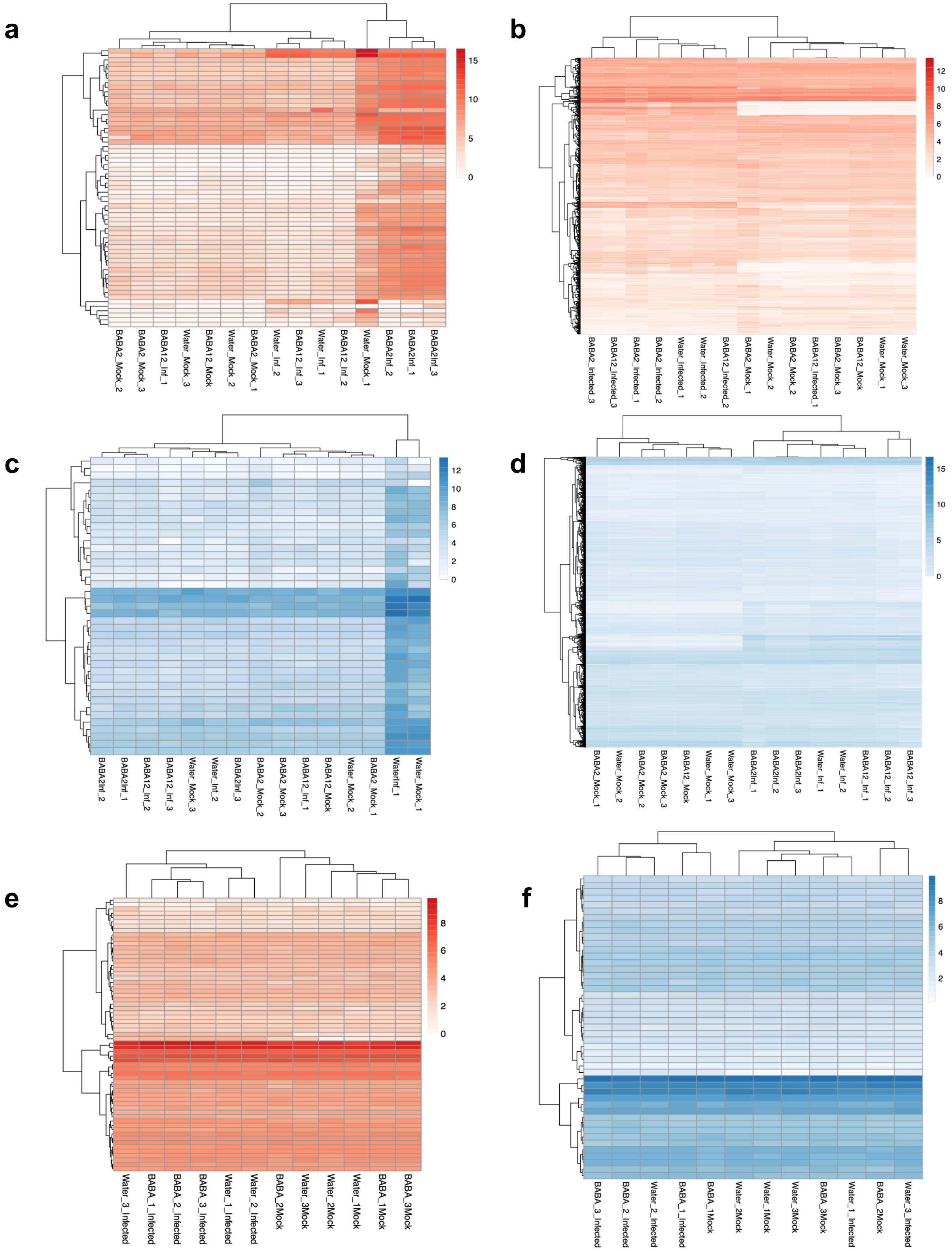

**Fig S5:** Expression of sRNA associated genes. Heatmap displaying normalised fragments per kilobase of transcript per million mapped reads (FPKM) value of genes containing a) upregulated diferentially expressed 24 nucleotide (nt) sRNA clusters conserved to heterograft (HG) and BABA graft (BG) phenotype. FPKM values display expression level in fruit tissue of mock and B. cinerea BABA2, Water and BABA12 plants. b) Upregulated differentially expressed 24 nt sRNA clusters in BG phenotype. FPKM values display expression level in fruit tissue of mock and B. cinerea infected BABA2, Water and BABA12 plants. c) downregulated diferentially expressed 24 nt sRNA clusters conserved to HG and BG phenotype. FPKM values display expression level in fruit tissue of mock and B. cinerea BABA2, Water and BABA12 plants. d) Downregulated diferentially expressed 24 nt sRNA clusters in BG phenotype. FPKM values display expression level in fruit tissue of mock and B. cinerea infected BABA2, Water and BABA12 plants. c) upregulated diferentially expressed 24 nt sRNA clusters conserved to HG and BG phenotype. FPKM values display expression level in leaf tissue of mock and B. cinerea BABA2, Water and BABA12 plants (T1). f) Downregulated diferentially expressed 24 nt sRNA clusters in BG phenotype. FPKM values display expression level in leaf tissue of mock and B. cinerea infected BABA2, Water and BABA12 plants (T1).
